# Engineering a Therapeutic Protein to Enhance the Study of Anti-Drug Immunity

**DOI:** 10.3390/biomedicines10071724

**Published:** 2022-07-18

**Authors:** Patricia E. Zerra, Ernest T. Parker, Wallace Hunter Baldwin, John F. Healey, Seema R. Patel, James W. McCoy, Courtney Cox, Sean R. Stowell, Shannon L. Meeks

**Affiliations:** 1 Center for Transfusion Medicine and Cellular Therapies, Department of Laboratory Medicine and Pathology, Emory University, Atlanta, GA 30322, USA; patricia.elizabeth.zerra@emory.edu (P.E.Z.); james.mccoy@emory.edu (J.W.M.); 2 Aflac Cancer and Blood Disorders Center, Children’s Healthcare of Atlanta, Department of Pediatrics, Emory University, Atlanta, GA 30322, USA; epark01@emory.edu (E.T.P.); hunterbaldwin@emory.edu (W.H.B.); jhealey@emory.edu (J.F.H.); seema.r.patel@emory.edu (S.R.P.); courtney.cox@emory.edu (C.C.); 3 Joint Program in Transfusion Medicine, Department of Pathology, Harvard Medical School, Boston, MA 02115, USA

**Keywords:** anti-drug antibodies, hemophilia A, factor VIII inhibitors, humoral immunity

## Abstract

The development of anti-drug antibodies represents a significant barrier to the utilization of protein-based therapies for a wide variety of diseases. While the rate of antibody formation can vary depending on the therapeutic employed and the target patient population receiving the drug, the antigen-specific immune response underlying the development of anti-drug antibodies often remains difficult to define. This is especially true for patients with hemophilia A who, following exposure, develop antibodies against the coagulation factor, factor VIII (FVIII). Models capable of studying this response in an antigen-specific manner have been lacking. To overcome this challenge, we engineered FVIII to contain a peptide (323–339) from the model antigen ovalbumin (OVA), a very common tool used to study antigen-specific immunity. FVIII with an OVA peptide (FVIII-OVA) retained clotting activity and possessed the ability to activate CD4 T cells specific to OVA_323–339_ in vitro. When compared to FVIII alone, FVIII-OVA also exhibited a similar level of immunogenicity, suggesting that the presence of OVA_323–339_ does not substantially alter the anti-FVIII immune response. Intriguingly, while little CD4 T cell response could be observed following exposure to FVIII-OVA alone, inclusion of anti-FVIII antibodies, recently shown to favorably modulate anti-FVIII immune responses, significantly enhanced CD4 T cell activation following FVIII-OVA exposure. These results demonstrate that model antigens can be incorporated into a therapeutic protein to study antigen-specific responses and more specifically that the CD4 T cell response to FVIII-OVA can be augmented by pre-existing anti-FVIII antibodies.

## 1. Introduction

The development of protein replacement therapy completely revolutionized the practice of medicine. This approach has allowed bioengineering strategies to leverage existing protein functions to replace defective proteins or supplement compromised physiologic pathways by use of novel protein-based approaches to treat a variety of disease states [1,2,3,4,5,6,7]. While most protein-based therapeutics are antibody-based and, thus, may not be considered to be as highly immunogenic, some therapeutics represent replacement of inadequately synthesized, dysfunctional, or completely absent protein products [7,8,9]. For patients completely deficient in a key protein in need of replacement, immunological tolerance may not develop, putting these individuals at risk of developing anti-protein antibodies following therapeutic exposure [10]. While immune responses to protein-based therapies can result in life-threatening anaphylactic reactions following re-exposure [11,12], most immune responses result in IgG antibody formation that interferes with the activity of the protein-based drug, rendering the therapeutic strategy ineffective [13,14]. While bypassing agents may exist for some diseases [15,16,17], this type of an immune response reduces effective therapeutic options for a given patient [18,19,20,21]. As a result, anti-drug antibodies can represent a critical barrier to the use of protein-based therapies in patients.

Among anti-drug antibodies directed against protein-based therapeutics, perhaps the most recognized example is the development of antibodies against the coagulation factor, factor VIII (FVIII) [22,23]. FVIII replacement therapy has been used for decades to prevent and treat bleeding episodes in patients with hemophilia A who are prone to uncontrolled bleeding secondary to reduced or absent FVIII levels. As one of the oldest examples of protein-based replacement therapy, initial FVIII treatment was provided in the form of cryoprecipitate generated from donated plasma. This was followed by the development of methods to purify FVIII as a plasma-derived resource [24]. Infectious disease exposure due to the use of plasma-derived FVIII products in addition to possible shortages in plasma donations, as recently experienced during the COVID-19 pandemic [25,26,27], motivated the development of recombinant FVIII (rFVIII) products, which now dominate FVIII replacement therapy. The rFVIII products include full-length (FL) and B domain deleted (BDD) rFVIII products, which have been shown to be equally efficacious and immunogenic. Despite the long history of FVIII use, anti-FVIII antibody development continues to plague patients with hemophilia A [18,28,29,30,31,32,33]. These antibodies can bind to FVIII, inhibit its activity, and therefore reduce or eliminate the therapeutic benefit of FVIII infusion [18,28,29,30,31,32,33,34,35,36]. While alternative strategies that can achieve baseline hemostasis in patients with hemophilia A have recently been developed that largely eliminate the need for continual FVIII replacement [15,16,17], bleeding episodes are still most effectively treated with FVIII [37,38,39]. As a result, while a variety of studies have sought to define key factors that influence anti-FVIII antibody formation [40,41,42,43,44,45,46,47,48,49], a greater understanding of the immune response to FVIII is needed if the deleterious consequences of anti-FVIII antibody formation are to be eliminated.

A critical component of sustained clinically relevant anti-drug antibody formation is the activation of CD4 T cells. CD4 T cell activation optimally occurs following antigen uptake by an activated antigen presenting cell (APC), necessitating not only efficient antigen degradation and presentation but also the appropriate activation of APCs prior to CD4 T cell exposure [50]. Activated and proliferated CD4 T cells are then capable of directly engaging antigen-specific B cells, where they can induce efficient class switching to IgG and facilitate the formation of long-lived plasma cells that sustain IgG production after antigen exposure [51]. In the absence of a robust CD4 T cell response, IgM may form. However, IgM that develop in this setting are often short-lived and low affinity, making them less likely to interfere with drug activity in the same way that IgG can following drug re-exposure [51]. Thus, the CD4 T cell response, while not directly responsible for antibody formation itself, plays a critical role in the production of clinically relevant antibody formation that interferes with the intended use of protein-based therapeutics.

Unlike models of infectious disease, vaccine development and even transfusion and both hematopoietic stem cell and solid organ transplantation [52,53,54,55], the immune response to protein-based therapeutics has been more difficult to define. While a variety of tools have been developed to aid in the study of the immune response in the setting of infectious disease or transplantation [56,57], a similar array of immunological tools to study the immune response to therapeutic proteins, including FVIII, has not been similarly available. This has especially been true for the evaluation of CD4 T cells, which are critical players in the development of an IgG response against a variety of (glyco)protein-based antigens, including FVIII and other protein-based therapeutics [58,59,60]. Unlike antibody responses themselves, where antibody levels can be determined by using the target antigen itself as the substrate for detection, CD4 T cell responses are restricted by the protein-derived peptides present in major histocompatibility complex class II (MHC II) molecules [61]. As protein degradation can result in the generation of many different peptide fragments and MHC II are highly polymorphic, the types of peptides presented following exposure to a given antigen can be difficult to predict, and therefore often requires empirical determination [62]. Even when CD4 T cell reactive peptides are identified, the ability to detect rapid T cell proliferation in response to antigen exposure is greatly facilitated by using T cell receptor (TCR) transgenics [63,64], which express a TCR that recognizes a given peptide–MHC complex.

Given the challenges associated with identifying CD4 T cell peptides presented following antigen exposure, coupled with the significant resources required to develop and validate a TCR transgenic, most studies have instead leveraged existing tools designed to evaluate the immune response to a model antigen by incorporating the antigen into the overall immunogen studied. This approach has been used for decades to study the immune response to viruses, bacteria, transplanted tissue and transfusion of blood products [60,65,66,67,68]. In each setting, despite the similar nature of the antigen targeted, the observed immune response appears to largely be governed by the vehicle containing the model antigen [60,65,66,67,68]. However, while this strategy has been employed in a variety of settings and has provided important insight into immunology in general, similar strategies have not been employed to define the CD4 T cell response to protein-based drug therapies. To overcome this challenge, we engineered a peptide (323–339) from the model antigen ovalbumin (OVA) into FVIII to generate FVIII-OVA. FVIII-OVA retained its clotting activity and equally important was able to induce anti-FVIII antibodies. TCR transgenics specific for the 323–339 OVA peptide (OTII) proliferated in vitro following incubation with APCs and FVIII-OVA. In contrast, very little OTII proliferation could be detected following exposure to FVIII-OVA in vivo. However, exposure to FVIII-OVA in the face of anti-FVIII antibodies induced significant OTII proliferation, suggesting that pre-existing antibodies may facilitate early CD4 T cell activation and additional alloantibody formation following FVIII-OVA exposure.

## 2. Materials and Methods

### 2.1. Construction, Expression and Purification of FVIII-OVA (RENeo FVIII with R740A Mutation)

Using the RENeo/BDD FVIII construct [69,70,71], the OVA consensus sequence (ISQAVHAAHAEINEAGR) and an R740A mutation was introduced using the PCR primer 5′ATCTGACTGAAGAGTCGTACGAGTTATTTCTCTGCCTGCTTCATTGATTTCTGCATGTGCTGCATGGACAGCTTGAGATATCGCAGGTTCAATGGCATTGTTTTTACTCAG-3′ (R740A mutation underlined). This was subcloned into the BSiWI/SACII restriction sites of RENeo/BDD. The resulting product, BDD human FVIII-OVA, was expressed in BHK-derived cells. The conditioned media was collected daily and the expressed proteins were purified following chromatography, as previously described [69]. Proteins were buffer exchanged and concentrated against 20 mM HEPES buffer at pH 7.4 containing 150 mM NaCl and 5 mM CaCl_2_ through 0.22 μm Millex^®®^ GP filter (Millipore, Carrigtwohill, Co., Cork, Ireland), as previously described [72].

### 2.2. Activation of FVIII by Thrombin and SDS-PAGE of FVIII

FVIII (BDD-FVIII or FVIII-OVA) was incubated with 0.6 units (U) of thrombin and HEPES buffered saline (HBS) for 30 min at room temperature. Thrombin activated samples or samples with HBS alone were then run on a 4–15% SDS-PAGE gel. For reducing, (beta-mercaptoethanol (BME) and Laemmli Sample Buffer (Biorad, Hercules, CA, USA) were used. FVIII-OVA was made as described above and BDD-FVIII was generated, as previously described [70,71].

### 2.3. Coagulation Assay

FVIII-OVA and BDD-FVIII were tested for factor VIIIa activity in a one-stage clotting assay using the STart Coagulation Analyzer (Diagnostica Stago, Asnieres, France) with human FVIII-deficient plasma used as a substrate, as described previously [73]. Briefly, samples containing BDD-FVIII or FVIII-OVA were incubated for 5 min at 37 °C with activated partial thromboplastin and human FVIII-deficient plasma, followed by the addition of 20 mM CaCl_2_. Time to fibrin clot formation was measured viscometrically. Pooled normal citrated human plasma (Factor Assay Control Plasma (FACT, George King Biomedical, Inc., Overland Park, KS, USA) served as a control for FVIII activity comparison. FVIII deficient plasma (severe hemophilia A plasma) and pooled normal plasma were obtained from George King Biomedical, Inc.

### 2.4. Mice

Initial immunogenicity experiments with FVIII-OVA were completed in E16 hemophilia A mice [74] that contain a neomycin cassette in exon 16 of the *F8* gene, resulting in a truncated FVIII protein, and are on a mixed C57BL/6(B6)/S129 background. Once available, FVIII knockout mice (hemophilia A mice, TKO) on a B6 background were used for all experiments [75]. TKO mice possess a deletion of the entire *F8* coding sequence [75], do not have any detectable *F8* messenger RNA, exhibit a bleeding phenotype, and develop anti-FVIII antibody titers following rFVIII exposure [76]. OTII × Thy 1.1 mice, which are also on a B6 background and possess a TCR specific to OVA_323–339_, were used for splenocyte isolation and adoptive transfer for tracking of FVIII-OVA-specific CD4 T cells [64,68]. HOD mice that express the HOD (hen egg lysozyme fused to OVA and human Duffy) antigen on red blood cells (RBCs) were used as donors [60,77,78,79,80,81,82]. B6 recipient mice were purchased from The Jackson Laboratory (Bar Harbor, ME, USA). Eight-to-twelve-week-old male and female mice were used. All animals were housed and bred in cages at the Emory University Department of Animal Resources facilities, and all experiments were performed under animal protocols approved by the Institutional Animal Care and Use Committee of Emory University.

### 2.5. OTII Splenocyte Labeling, Adoptive Transfer and Analysis of CD4 T Cell Proliferation In Vitro and In Vivo

Splenocytes from OTII mice that express a TCR specific to OVA_323–339_ were isolated and labeled with carboxyfluorescein succinimidyl ester (CFSE) (ThermoFisher, Waltham, MA, USA), which was confirmed by flow cytometry. Adoptive transfer of 10 × 10^6^ CFSE-labeled OTII × Thy1.1 splenocytes was performed via retro-orbital injection 24 h prior to saline, FVIII-OVA, or HOD transfusion. For in vitro analysis, CFSE-labeled splenocytes from OTII × Thy1.2 mice were cultured with FVIII-OVA for 7 days. Cells were then stained with Brilliant Violet 450 anti-mouse CD4 and APC anti-mouse CD8, and proliferation was evaluated by flow cytometry. To evaluate proliferation of Thy 1.1 CD4 T cells in vivo, splenocytes were isolated and stained with Live/Dead Zombie NIR, Brilliant Violet 786 anti-mouse CD3, V500 anti-mouse CD4, and APC anti-mouse Thy1.1 [60]. Samples were run on a Northern Lights (Cytek Biosciences, Bethesda, MD, USA) spectral flow cytometer or an LSR-II (BD Biosciences, Franklin Lakes, NJ, USA) flow cytometer and analyzed using FlowJo software, version 10 (BD, Ashland, OR, USA).

### 2.6. RBC Isolation and Transfusion

RBCs were collected from HOD mice into acid citrate dextrose (ACD) and washed 3 times in 1 × phosphate-buffered saline (PBS). B6 mice were transfused with 50 μL of packed RBCs diluted in PBS to 300 μL total volume via lateral tail vein injection [60,68,77,78,81,83,84,85,86].

### 2.7. FVIII Administration, Plasma Collection and Analysis of Anti-FVIII Antibodies

BDD-FVIII, FL-rFVIII or FVIII-OVA in a 100 μL total volume of sterile saline was administered weekly for 5–8 weeks via retro-orbital injection. Weight-based dosing (10–25 U/kg) or standard dosing (1–2 μg) was used. Within individual experiments, dosing was determined based on molar equivalents of each FVIII product. To examine anti-FVIII inhibitor formation in hemophilia A mice following exposure to FVIII, blood was collected from the orbital venous plexus with heparinized capillary tubes 7 days after the last injection of FVIII. A sandwich enzyme-linked immunosorbent assay (ELISA) was used for measuring anti-FVIII IgG and Bethesda assay was used for the measurement of FVIII inhibitor titers, as previously described [41,76,80,87,88,89].

### 2.8. Statistical Analysis

A Mann–Whitney U test or 1-way analysis of variance (ANOVA) test with a post hoc Tukey multiple-comparisons test was performed to determine the significance of results. Prism, version 9 (GraphPad Software, La Jolla, CA, USA) was used to perform all statistical analyses. *p* values <0.05 were considered statistically significant.

## 3. Results

While a variety of model antigens exist that can be coupled to distinct immunogens, OVA is an efficiently processed model antigen that is well-established as an immunological tool for characterizing CD4 T cell immunity and used in the commonly employed B6 recipient background. As most studies aimed at studying anti-FVIII antibody development have used FVIII-deficient hemophilia A mice on a B6 background and the genetic tools available are most commonly on this murine background, we elected to incorporate OVA into FVIII in an effort to track antigen-specific CD4 T cell responses following FVIII exposure in vivo.

**Figure 1:**
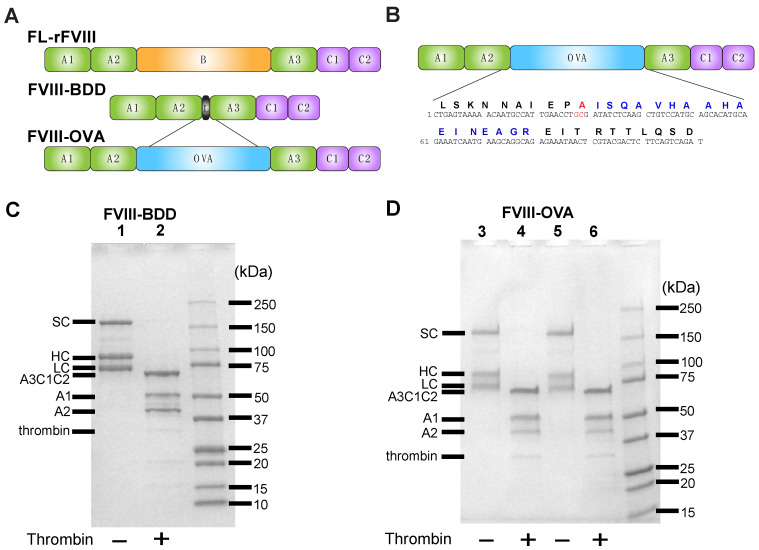
FVIII-OVA design and cleavage by thrombin. (**A**) Representative diagram showing FL-rFVIII, B-domain deleted (BDD) FVIII and the insertion of ovalbumin (OVA) peptide into the B-domain of BDD-FVIII. A1, A2, A3, B, C1 and C2 = FVIII domains. L = linker peptide. OVA = OVA peptide 323–339. (**B**) Insertion site of R740A mutation (red), followed by OVA consensus sequence (blue). SDS-PAGE analysis of (**C**) BDD-FVIII and (**D**) FVIII-OVA (shown in duplicate). Purified rFVIII or FVIII-OVA protein was resolved by 9% SDS-PAGE under reducing conditions and visualized by Coomassie blue staining. BDD-FVIII (lanes 1,2) and FVIII-OVA (lanes 3–6) were treated with or without thrombin prior to SDS-PAGE. Polypeptides were identified as labeled: single chain (SC), heterodimeric heavy chain (HC), light chain (LC), light chain (A3-C1-C2) and thrombin-cleaved A1 and A2 fragments.

To engineer FVIII with the OVA peptide 323–339, we took advantage of very similar immunogenicity profiles of FL-rFVIII and BDD-FVIII by using a BDD-FVIII backbone and adding OVA_323–339_ in the location of the B domain to generate FVIII-OVA (Figure 1A,B). Similarly to BDD-FVIII (Figure 1C), purified FVIII-OVA (Figure 1D) migrated at the expected molecular weight as three distinct bands, including the expected single chain, heavy chain and light chain. Activation of FVIII requires cleavage by the protease thrombin, another key player in the clotting cascade. To determine whether FVIII-OVA retained sensitivity to thrombin cleavage, FVIII-OVA was incubated with thrombin, followed by SDS-PAGE analysis (Figure 1D). While the single chain, heavy chain and light chains could be readily detected prior to thrombin cleavage, incubation with thrombin resulted in migration of each band as predicted, in addition to the formation of A1 and A2 fragments [90,91]. A faint thrombin band, which migrates at 32.5 kDa, can likewise be detected in thrombin treated samples. These results demonstrate that OVA can be incorporated into FVIII, but that doing so does not appear to affect its sensitivity to cleavage by thrombin.

Although conflicting data exist regarding the possible role of the clotting activity of FVIII in the development of an immune response, in order to recapitulate the intent and purpose of FVIII exposure and, therefore, possible immune factors that may be engaged following FVIII infusion, we next examined whether FVIII-OVA can facilitate clot formation using a commonly employed clotting assay used clinically to measure clotting factor activity. FVIII-OVA demonstrated specific FVIII activity of 6793 IU FVIII/mg protein. This was similar clotting activity when compared to BDD-FVIII [92] (8329 IU FVIII/mg protein). Taken together, these results demonstrate that FVIII-OVA is not only sensitive to thrombin, but that FVIII dependent clotting can occur with the same efficiency as observed following the use of BDD-FVIII, which has been used in the study of anti-FVIII antibody formation in pre-clinical models.

**Figure 2:**
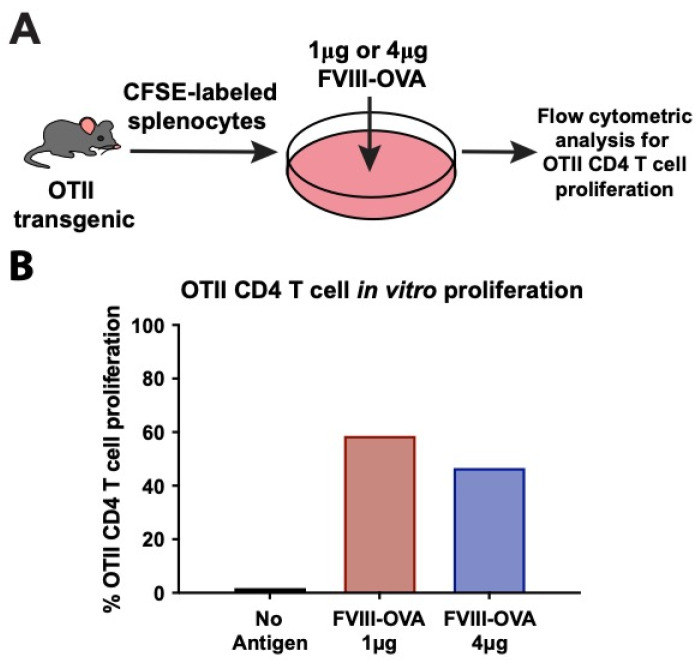
FVIII-OVA CD4 T cell epitope is functional in vitro. (**A**) Splenocytes from OTII transgenic mice were CFSE-labeled, then cultured for 7 days either with no antigen or with 1 or 4 μg FVIII-OVA in vitro. OTII CD4 T cell proliferation was assessed by flow cytometry. (**B**) Quantification of % OTII CD4 T cell proliferation with no antigen, or 1 μg or 4 μg FVIII-OVA. Plotted % is the average of 2–3 individual samples.

As the purpose of engineering FVIII with OVA_323–339_ was to facilitate the detection of an antigen-specific CD4 T cell response following FVIII exposure, we next determined whether OVA_323–339_ within FVIII can induce CD4 T cell proliferation in vitro using CD4 T cells isolated from TCR transgenics specific to OVA_323–339_ (OTIIs). This was accomplished by incubating FVIII-OVA with CFSE-labeled OTII splenocytes containing APCs to facilitate the detection of OTII CD4 T cell proliferation in response to FVIII-OVA (Figure 2A). Using this approach, OTII proliferation could be observed as a stepwise decrease in CFSE fluorescence, as each daughter cell contains less CFSE labeled contents. While incubation of OTII splenocytes in the absence of FVIII-OVA failed to result in any change in the fluorescence of OTIIs, inclusion of FVIII-OVA resulted in an increased number of CD4 T cells detected with diminished levels of CFSE, indicating proliferation in response to FVIII-OVA exposure (Figure 2B). These results suggest that following exposure to an APC, this construct can be taken up, processed and presented in such a way that OTII CD4 T cells can respond to the OVA peptide within the overall protein.

**Figure 3:**
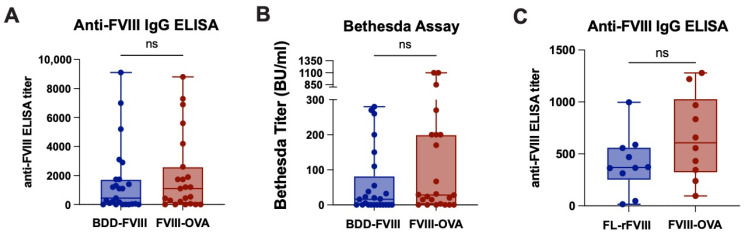
FVIII-OVA is immunogenic in mice with hemophilia A. Hemophilia A mice received weekly (molar equivalent) doses of BDD-FVIII, FL-rFVIII or FVIII-OVA. Plasma was collected one week after the final injection and was evaluated for anti-FVIII IgG by ELISA (**A**,**C**) and for inhibitors by Bethesda assay (**B**). Statistics were generated using a Mann–Whitney U test. Error bars indicate standard deviation. Horizontal lines represent mean of each group. ns = not significant.

As the ultimate goal of generating FVIII-OVA was to engineer a therapeutic protein construct with an embedded model antigen capable of defining the antigen-specific CD4 T cell response following FVIII exposure in vivo, we next sought to use FVIII-OVA to measure responsiveness of OVA-specific CD4 T cells following FVIII-OVA infusion. To accomplish this, we first sought to determine whether FVIII-OVA can induce an anti-FVIII antibody response following infusion. To this end, E16 hemophilia A recipients were either exposed to FVIII-OVA or BDD-FVIII, followed by evaluation of anti-FVIII antibody formation, as done previously [41,80]. Exposure to FVIII-OVA induced a robust anti-FVIII antibody response that was very similar to the immune response observed following exposure to BDD-FVIII (Figure 3A). Furthermore, similar Bethesda titers were observed, suggesting that FVIII-OVA exposure results in comparable levels of inhibitory antibody production as BDD-FVIII exposure (Figure 3B). Given that our classically used E16 mice are on a mixed B6 and S129 background and the OTII × Thy1.1 mice are on a B6 genetic background, we needed a hemophilia mouse model on a B6 background to ensure results were not impacted by strain differences. With the development of the TKO hemophilia A mice on a B6 background, we repeated the immunogenicity experiment using either FL-rFVIII or FVIII-OVA. We confirmed that a similar robust anti-FVIII antibody response was measured in both groups (Figure 3C). These results suggest that FVIII-OVA retains the ability to induce anti-FVIII antibody formation in this pre-clinical model.

**Figure 4:**
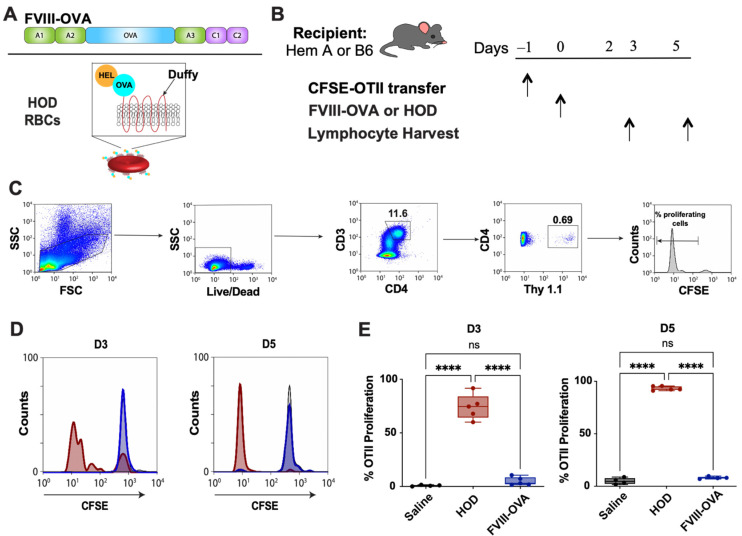
FVIII-OVA does not induce OTII CD4 T cell proliferation in vivo following one exposure. (**A**) Diagrams showing presence of OVA peptide in FVIII (FVIII-OVA) and the HOD (hen egg lysozyme [HEL] and OVA fused with the human RBC antigen Duffy). RBC model system. (**B**) Hemophilia A or B6 mice were adoptively transferred with 10 × 10^6^ CFSE-labeled OTII splenocytes, followed by a single infusion of saline, 2 μg FVIII-OVA or HOD RBCs 24 h later. Lymphocytes were harvested on days 3 and 5 following antigen exposure, and examined by flow cytometry. (**C**) Gating strategy showing assessment of OTII CD4 T cell proliferation. Representative histogram (**D**) and quantification (**E**) of OTII CD4 T cell proliferation in mice treated with saline (black), HOD RBCs (red) or FVIII-OVA (blue). Error bars represent standard deviation, **** = *p* < 0001; 1-way ANOVA with Tukey’s post hoc test. D3 = day 3; D5 = day 5; ns = not significant.

Given the similar ability of FVIII-OVA to induce anti-FVIII antibodies following infusion in vivo, coupled with its ability to induce OTII proliferation following incubation with APCs in vitro, we next sought to leverage this tool to define the antigen-specific CD4 T cell response to FVIII-OVA in vivo. To accomplish this, we adoptively transferred CFSE-labeled OTII CD4 T cells into hemophilia A (TKO) recipients, followed by exposure to FVIII-OVA (Figure 4A,B). Unexpectedly, despite the ability of FVIII-OVA to induce CD4 T cell proliferation in vitro, very little, if any, detectable OTII proliferation was observed following FVIII-OVA injection in vivo (Figure 4C–E). Lack of proliferation did not appear to reflect a defect in the ability of CFSE-labeled CD4 OTIIs to proliferate in vivo, as exposure of B6 recipients that likewise received OTIIs, followed by transfusion of RBCs that express a chimeric antigen of hen egg lysozyme, OVA and the human Duffy antigen (HOD), resulted in rapid OTII proliferation when evaluated in parallel (Figure 4D,E).

**Figure 5:**
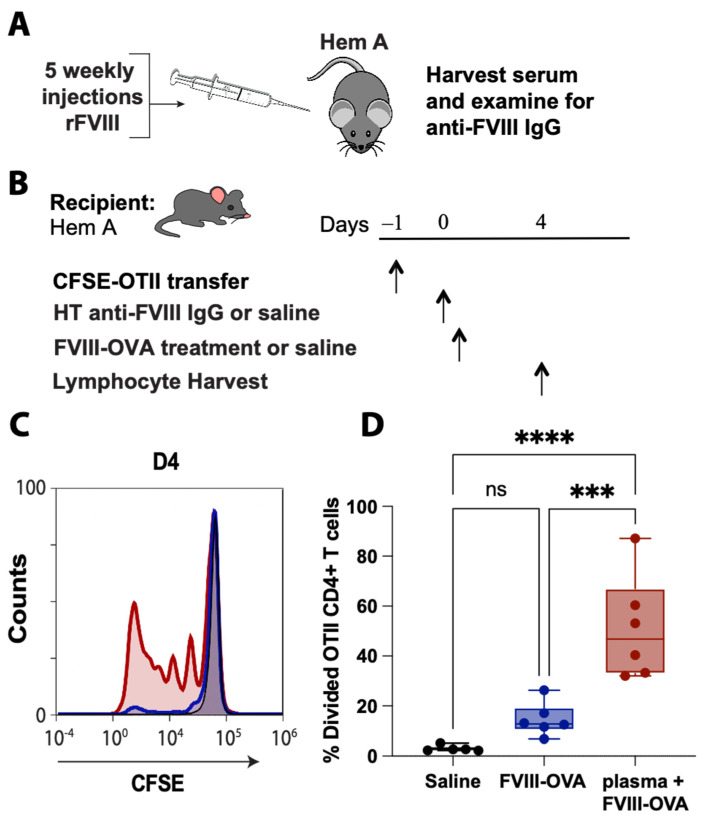
FVIII-OVA induces OTII CD4 T cell proliferation following passive immunization with plasma containing anti-FVIII IgG. (**A**) Hemophilia A mice received 5 weekly injections of rFVIII, followed by serum collection and ELISA to detect anti-FVIII IgG. (**B**) Hemophilia A mice were adoptively transferred with 10 × 10^6^ CFSE-labeled OTII splenocytes. Twenty-four hours later, mice were infused with either saline, 2 μg FVIII-OVA, or 200 μL pooled high-titer (HT) serum plus 2 μg FVIII-OVA 2–4 h later. Lymphocytes were isolated 4 days later and assessed by flow cytometry. Representative histogram (**C**) and quantification (**D**) of OTII CD4 T cell proliferation in mice treated with saline (black), FVIII-OVA (blue) and HT plasma plus FVIII-OVA (red). Error bars represent standard deviation, **** = *p* < 0.0001, *** = *p* < 0.0007; 1-way ANOVA with Tukey’s post hoc test. HT = high-titer, D4 = day 4; ns = not significant.

Recent studies suggest that low levels of anti-FVIII antibodies may augment an immune response to FVIII [93], suggesting that some level of pre-existing immunity may be required for an efficient CD4 T cell response to be realized following FVIII exposure. This may especially be important when considering that OVA_323–339_ within FVIII-OVA is an artificial construct within a single protein, as opposed to an entire cell or infectious organism and that the efficiency of processing and presenting OVA_323–339_ within FVIII-OVA may therefore differ from other model systems where OVA has been used as a surrogate for studying antigen-specific immune responses. To determine whether inclusion of anti-FVIII antibodies may enhance the efficiency of OTII proliferation following FVIII-OVA exposure, we next adoptively transferred CFSE-labeled OTIIS, followed by passive administration of anti-FVIII antibodies and infusion of FVIII-OVA (Figure 5A,B). In contrast to the lack of significant proliferation observed following FVIII-OVA administration alone, OTII proliferation could be readily observed in anti-FVIII antibody passively immunized recipients (Figure 5C,D). These results demonstrate that OVA_323–339_ can induce proliferation of CD4 T cells following FVIII-OVA infusion in vivo, but that a variety of factors, including the presence of anti-FVIII antibodies, may be important in dictating the ability of OTIIs to readily detect and respond to OVA_323–339_ following FVIII-OVA exposure.

## 4. Discussion

The development of anti-drug antibodies represents a significant barrier to the use of protein-based therapeutics in the treatment of disease [13,14]. However, unlike the immune responses observed following infection, transplantation or transfusion, the mechanisms responsible for anti-drug antibody formation have remained relatively unexplored. The lack of understanding regarding anti-drug antibody formation in part stems from inadequate tools capable of leveraging existing strategies designed to evaluate antigen-specific responses following protein-based therapy exposure. The results of the present study suggest that incorporation of a model antigen into FVIII and perhaps other therapeutic proteins may provide a useful strategy when seeking to study CD4 T cell responses generated following therapeutic delivery of protein-based therapies.

Anti-drug antibody formation in general represents a unique form of immunity. Traditionally, antibody formation following exposure to antigen only occurs in the presence of an adjuvant, which is responsible for activating a series of pathways that ultimately converge to facilitate APC activation and CD4 T cell proliferation in response to a (glyco)protein antigen [94,95,96,97,98,99,100,101]. The need for appropriate APC activation following antigen exposure is most apparent when considering the requirement of adjuvant for vaccine responses, where various adjuvants are used to ensure an adequate immune response following exposure to distinct antigens designed to induce protective immunity in vaccinated individuals [100]. In contrast, microbes themselves possess pathogen associated molecular patterns (PAMPs) that are detected by hard-wired innate immune receptors that alert the immune system that antigen exposure may be occurring in the context of infection [95,96,101]. While vaccine adjuvants are designed to leverage these innate immune activating programs, protein-based therapeutics are not known to intrinsically express motifs that mimic PAMPS or otherwise engage innate immune activation pathways to facilitate immunity [102]. Consistent with this, most individuals do not generate anti-drug antibodies following exposure [102].

Given the lack of known PAMPs in protein-based therapies, the mechanisms whereby a protein can induce an immune response in the absence of a clear adjuvant has remained somewhat elusive. Several studies have suggested that inflammatory cues at the time of delivery of protein-based therapies may serve as a surrogate for PAMP exposure [41]. Inflammation in response to infection, injury or other sources may activate APCs and other key immune cells, thereby priming the immune system to generate an immune response following exposure to therapeutic proteins such as FVIII [103]. However, additional recipient variables may also play a role, including genetic polymorphisms that increase the likelihood of an immune response in general and an optimal repertoire of MHC molecules capable of presenting peptides derived from the antigen for appropriate CD4 T cell help [22]. Immune responsiveness to a protein-based therapy may therefore represent a continuum of variables, from underlying genetic deposition at baseline to inflammatory cues at the time of administration that may ultimately converge to dictate the likelihood that an immune response occurs following exposure.

A critical component of sustained clinically relevant anti-drug antibody formation is the activation of CD4 T cells. CD4 T cell activation optimally occurs following antigen uptake by an activated APC, necessitating not only efficient antigen degradation and presentation but also the appropriate activation of APCs prior to CD4 T cell exposure [50]. Activated and proliferated CD4 T cells are then capable of directly engaging antigen-specific B cells, where they can induce efficient class switching to IgG and facilitate the formation of long-lived plasma cells that sustain IgG production after antigen exposure [51]. In the absence of a robust CD4 T cell response, IgM may form. However, IgM that develop in this setting are often short-lived and low affinity, making them less likely to interfere with drug activity in the same way that IgG can following drug re-exposure [51]. Thus, the CD4 T cell response, while not directly responsible for antibody formation itself, plays a critical role in the production of clinically relevant antibody formation that interferes with the intended use of protein-based therapeutics.

Despite the importance of CD4 T cells in regulating the development of anti-drug antibodies, the examination of a CD4 T cell response can be challenging in model systems. When examining an antibody response, the drug itself can serve as the antigen target and numerous approaches, including the use of ELISA-based strategies, can be employed to detect possible antibody formation. Thus, antibody formation can be detected against nearly any antigen examined. In contrast, as CD4 T cell responses require the presentation of peptide fragments of the protein-based therapeutic itself, the actual protein fragments involved in a robust CD4 T cell response must be identified [61,62]. Often, the optimally presented peptide fragments may differ depending on the MHC repertoire of the recipient, making it difficult to develop a uniform approach to studying antigen-specific CD4 T cell responses in vivo [61,62]. To overcome these limitations, TCR transgenics were generated based on well-characterized TCRs known to respond to a defined peptide fragment of a given antigen [63,64]. As the immune response to model antigens such as OVA had been well characterized, this and related antigens quickly became the targets of developing TCR transgenics. Using this approach, a T cell of known specificity can be evaluated for responsiveness in vivo following exposure to the model antigen. This contrasts with what can be performed in vitro, where the entire antigen can be used to generate a T cell response, but where the response observed may or may not reflect what happens after actual exposure in vivo. Given the challenges and significant resources required to generate and validate TCR transgenics, most studies have instead incorporated model antigens into a target immunogen of interest, allowing pre-existing TCR transgenic tools to be used when studying the immune response to a given target. The use of this strategy here highlights that this same approach can be applied to therapeutic proteins and suggests that incorporation of OVA or related model antigens in therapeutic protein-based drug targets may be useful when seeking to examine the CD4 T cell response following therapeutic protein exposure.

The inability of FVIII-OVA to initially generate a robust CD4 T cell response in vivo was unexpected and illustrates the importance of this model in defining key factors that may drive T cell responses following protein-based drug delivery. One possibility for lack of significant in vivo response to a single dose of FVIII-OVA is the much lower dose of OVA peptide when FVIII-OVA is given at therapeutic doses of FVIII compared to the amount of OVA in a HOD RBC transfusion. Recent studies suggest that pre-existing antibodies, most notably against xenoantigen carbohydrates on some FVIII products, may enhance antibody formation following FVIII exposure [76], raising the possibility that existing anti-FVIII antibodies may likewise facilitate the formation of anti-FVIII antibodies following re-exposure. While FVIII-unexposed hemophilia A mice do not appear to possess detectable anti-FVIII antibodies, healthy patients have been shown to exhibit low levels of anti-FVIII antibodies [104,105], although the possible influence of these antibodies on the likelihood of de novo anti-FVIII antibody formation remains relatively unexplored. To explore the possible influence of pre-existing anti-FVIII antibodies on CD4 T cell activation, recipients were given anti-FVIII antibodies, followed by FVIII-OVA exposure. In contrast to the lack of detectable CD4 T cell proliferation observed following infusion of FVIII-OVA alone, OTII proliferation was readily detected in recipients previously exposed to anti-FVIII antibodies. While the mechanism whereby pre-existing antibodies facilitate T cell proliferation remains to be explored and is certainly beyond the scope of the present study, rapid APC uptake of antigen, which may facilitate APC activation in the absence of adjuvant, in addition to the possible ability of antibody to retain FVIII-OVA in the B cell follicle, represent just a few of the possible mechanisms whereby antibodies may facilitate CD4 T cell responses following antigen exposure.

As with any study, this study is not without limitations. Whether the response to FVIII-OVA is representative of the T cell response to FVIII itself remains to be determined. Defining such a response will require the development of a TCR transgenic that targets a FVIII-specific peptide using the same pre-clinical model. However, given the resources required to develop a TCR transgenic system, such a comparison is beyond the scope of the present study. When evaluated in parallel, it is important to note that HOD RBCs alone were able to induce OTII responses following initial alloantigen exposure, demonstrating that OTII responses can be observed following exposure to an intravascular antigen in the absence of pre-existing antibodies. It is also possible that the overall immune response to FVIII-OVA fundamentally differs from FVIII itself. However, when used in other contexts, the OVA response largely adopts the overarching immune response observed following exposure in the absence of OVA [60,65,66,67,68], suggesting that that vehicle carrying the OVA drives the nature of the immune response more than OVA itself.

In conclusion, these studies illustrate that a model antigen can be incorporated into a therapeutic protein and used to study CD4 T cell responses by leveraging pre-existing immunological tools. This proof of principle can therefore be utilized in future studies to further optimize FVIII-OVA constructs and moreover serve as a template for the incorporation of OVA or other model antigens into additional therapeutic proteins to facilitate study of the immune response following protein-based therapeutic delivery.
